# Correlates of Health-Related Social Media Use Among Adults

**DOI:** 10.2196/jmir.2297

**Published:** 2013-01-30

**Authors:** Rosemary Thackeray, Benjamin T Crookston, Joshua H West

**Affiliations:** ^1^Brigham Young UniversityDepartment of Health ScienceProvo, UTUnited States

**Keywords:** social media, Internet, health information, consumer

## Abstract

**Background:**

Sixty percent of Internet users report using the Internet to look for health information. Social media sites are emerging as a potential source for online health information. However, little is known about how people use social media for such purposes.

**Objectives:**

The purpose of this study was two-fold: (1) to establish the frequency of various types of online health-seeking behaviors, and (2) to identify correlates of 2 health-related online activities, social networking sites (SNS) for health-related activities and consulting online user-generated content for answers about health care providers, health facilities, or medical treatment.

**Methods:**

The study consisted of a telephone survey of 1745 adults who reported going online to look for health-related information. Four subscales were created to measure use of online resources for (1) using SNS for health-related activities; (2) consulting online rankings and reviews of doctors, hospitals or medical facilities, and drugs or medical treatments; (3) posting a review online of doctors, hospitals or medical facilities, and drugs or medical treatments, and (4) posting a comment or question about health or medical issues on various social media. Univariate and multivariate logistic regression analyses were performed.

**Results:**

Respondents consulted online rankings or reviews (41.15%), used SNS for health (31.58%), posted reviews (9.91%), and posted a comment, question, or information (15.19%). Respondents with a chronic disease were nearly twice as likely to consult online rankings (odds ratio [OR] 2.09, 95% CI 1.66-2.63, *P*<.001). Lower odds of consulting online reviews were associated with less formal education (OR 0.49, 95% CI 0.37-0.65, *P*<.001) and being male (OR 0.71, 95% CI 0.57-0.87, *P*<.001). Respondents with higher incomes were 1.5 times as likely to consult online rankings or reviews (OR 1.49, 95% CI 0.10-2.24, *P*=.05), than respondents with a regular provider (OR 2.05, 95% CI 1.52-2.78, *P*<.001), or living in an urban/suburban location (OR 1.61, 95% CI 1.17-2.22, *P*<.001). Older respondents were less likely to use SNS for health-related activities (OR 0.96, 95% CI 0.95-0.97, *P*<.001), as were males (OR 0.70, 95% CI 0.56-0.87, *P*<.001), whereas respondents with a regular provider had nearly twice the likelihood of using SNS for health-related activities (OR 1.89, 95% CI 1.43-2.52, *P*<.001).

**Conclusions:**

People are using social media for seeking health information. However, individuals are more likely to consume information than they are to contribute to the dialog. The inherent value of “social” in social media is not being captured with online health information seeking. People with a regular health care provider, chronic disease, and those in younger age groups are more likely to consult online rankings and reviews and use SNS for health-related activities.

## Introduction

The Internet is becoming an increasingly common source of health information. Approximately 60% of Internet users report using the Internet to look for health information [1,2]. In addition to seeking health information, Wen et al [3] found that 15% of Internet users also tracked personal health information on the Internet. Determinants of seeking health information online include education, gender, race, age, presence of children in the home, having a poor personal health condition, and geographic residence [1,4-7]. Similarly, predictors of using the Internet to track personal health information include gender, race, education, and having a health care provider [3].

Historically, online health seeking meant visiting an agency- or organization-sponsored website. Recently, social media sites are emerging as a potential source of online health information [8]. Social media refers to “activities, practices, and behaviors among communities of people who gather online to share information, knowledge, and opinions using conversational media” [9]. These social media are broadly categorized as forums and message boards, review and opinion sites, social networks (eg, Facebook), blogging and microblogging (eg, Twitter), bookmarking, and media sharing (eg, YouTube) [10].

Individual use of social media is steadily increasing. Nearly two-thirds (65%) of adult Internet users in the United States are involved with a type of social media called *social networking sites* (SNS), such as MySpace, Facebook, or LinkedIn [11]. Technorati currently registers over 1.3 million blogs [12], 13 percent of Internet users (140 million people) have a Twitter account [13,14], and Facebook has 955 million active users [15]. By 2015, it is estimated that the number of individuals and corporations who have social networking accounts will reach over 3 billion [16].

Social media and SNS use varies by demographics. There are statistically significant differences in SNS use between younger and older ages and between males and females [11]. However, SNS are used fairly equally across education, income, race/ethnicity, and rural and urban locations [11]. Chou and colleagues [17] found that age and education were predictors of 3 forms of social media use (ie, participating in online support groups, blogging, and visiting a SNS).

In contrast to going online to seek health information, social media technologies allow online social media users to create, distribute, and share information independent of an organization. The level of use and involvement with social media technologies varies by individual. Bernoff and Anderson [18] and Li and Bernoff [19] classify individuals based on how they use social media. These classifications, although not mutually exclusive, include creators, conversationalists, critics, collectors, joiners, or spectators. Similarly, Hoffman and Novak [20] identify 4 goals for social media use: create, connect, consume, and control. The main conclusion from both typologies is that the range of social media activities that people engage in varies from consuming to creating content.

Despite the near ubiquity of social media use and the high prevalence of health information seeking on the Internet, there is a dearth of literature about the characteristics of people who use social media for seeking health information and how these people engage with social media. Thus, additional research is needed to determine whether social media users are primarily spectators, or if they are creators or critics. That is, are they looking for information or are they becoming part of the information creation and sharing process? Knowing the correlates of social media use for health information can allow health professionals to more accurately segment populations and tailor interventions accordingly. Therefore, the aim of this research was two-fold. First, to establish the frequency of various forms (eg, spectators, creators, or critics) of online health-seeking behaviors. Second, this research seeks to identify correlates of 2 health-related online activities: (1) using SNS for health-related activities, and (2) consulting online user-generated content for answers about health care providers, health facilities, or medical treatment.

## Methods

### Data Source and Sample

The data for this study were taken from the 2010 Health Tracking Survey conducted by Princeton Survey Research on behalf of the Pew Internet & American Life Project [21]. The data were collected during August to September 2010 through a telephone survey that included both cell phones and landlines. A random digit method was used to select participants who were US residents, aged 18 years and older, and who spoke English (n=3001). Data were weighted to the most recent US Census Bureau’s Current Population Survey. Data were stripped of identifying information and made available to the public. For the current study, inclusion criteria were adults who used the Internet at least occasionally (Pew question Q6a) and who reported going online to look for health-related information (Pew question healthseek). The final sample size was 1745.

### Measures

#### Demographic, Socioeconomic, and Health Status

Demographic, socioeconomic, and health status covariates included ethnicity, education, income, gender, age, race, marital status, having a chronic health condition, geographic community type, health insurance status, and having a family doctor or health care professional. Response categories for race, education, and marital status were collapsed to account for small cell sizes.

To measure an individual’s level of social media health engagement, we created 4 subscales based on related survey items. Each of the response variables were dichotomous and coded as yes or no. We calculated Cronbach alpha to estimate internal reliability for each scale.

#### Used Social Networking Sites for Health-Related Activities

The 5 questions that focused on using SNS for health-related activities included (1) get health information, (2) start or join a health-related group, (3) follow your friend’s personal health experiences or health updates, (4) raise money or draw attention to a health-related issue or cause, and (5) remember or memorialize others who suffered from a certain health condition (Pew questions Q26a-e). The composite scale had an internal reliability of Cronbach alpha=.66

#### Consulted Online Rankings or Reviews

Three questions focused on consulting online rankings or reviews of (1) doctors or other providers, (2) hospitals or other medical facilities, and (3) particular drugs or medical treatments (Pew questions Q29a-c; Cronbach alpha=.69).

#### Posted a Review Online

Three questions focused on whether respondents had posted a review online of (1) a doctor, (2) a hospital, or (3) his/her experiences with a particular drug or medical treatment (Pew questions Q29d-f; Cronbach alpha=.61)

#### Posted a Comment or Question on Social Media

Five questions asked if respondents had posted comments, questions, or information about health or medical issues on various social media. These included (1) an online discussion, a listserv, or other online group forum, (2) a blog, (3) a social networking site, such as Facebook, MySpace, or LinkedIn, (4) Twitter or another status update site, and (5) a website of any kind, such as a health site or news site that allows comments and discussion (Pew questions Q25a-e; Cronbach alpha=.80)

### Data Analysis

Unadjusted univariate analyses of demographics, socioeconomic, and health status variables with each social media–health engagement scale were computed. Variables that were significantly associated with the dependent variable were included in a multivariate regression model. Multivariate logistic regression analysis was performed with social media health engagement as the dependent variable and the demographics, socioeconomic, and health status variables as covariates. All analyses were conducted using IBM SPSS Statistics version 20 (IBM Corp, Armonk, NY, USA).

## Results

### Demographic Characteristics

More than half of the study sample was female (56.16%, 980/1745) and white (79.20%, 1382/1745) (see Table 1). College graduates comprised 39.43% (689/1745) of the sample, 29.46% (514/1745) reported a household income between US $75,000 and $150,000, and 86.88% (1516/1745) reported having health insurance. Respondents reported consulting online rankings or reviews (41.15%, 718/1745) and using SNS for health (31.58%, 551/1745) more than they reported contributing content through posting reviews of doctors, hospitals, drugs, or medical treatments (9.91%, 173/1745), or posting a comment, question, or information about health or medical issues on a blog, SNS, Twitter, website, or online discussion or forum (15.19%, 265/1745).

### Correlates of Social Media Health Engagement and Regression Analyses

Regression analyses revealed few correlates for posting reviews of a doctor, hospital, drug, or medical treatment (chronic disease, income, age, health insurance) and for posting a comment, question, or information on various social media sites (chronic disease, age, marital status). Therefore, the further analysis and data presented here are limited to using SNS for health and consulting online rankings or reviews.

An examination of correlates of consulting online rankings or reviews identifies several factors that are associated with higher use of online rankings and reviews (see Table 2). For example, approximately half (49.27%, 339/688) of those with a college degree reported using online rankings or reviews compared with 40.71% (204/501) of those with some college and 31.50% (172/546) of those with a high school education or less. Factors associated with use of SNS for health included income, gender, age, marital status, and having a personal or family doctor or health care provider.

### Unadjusted Regression Analyses for Consulting Online Rankings

Unadjusted regression analyses revealed numerous factors associated with consulting online rankings or reviews of doctors, hospitals, drugs, or medical treatments (see Table 3). Having a chronic disease, reporting a higher annual income, living in an urban/suburban location, reporting health insurance coverage, and having a regular health care provider were each independently associated with increased odds of consulting online rankings. Decreased odds were observed among older respondents, those who were unmarried, those with lower levels of education, males, and those who were black/African American.

### Unadjusted Regression Analyses for Using Social Networking Sites for Health

Table 4 presents the results of the unadjusted regression analyses for using SNS for health-related activities, such as getting information, joining a group, following friends’ health experiences, raising money, increasing awareness, or remembering or memorializing others. Older respondents and males were each less likely to engage in such behaviors. Respondents who reported being unmarried or having a regular health care provider were more likely to use SNS for health-related purposes.

### Adjusted Regression Analyses for Consulting Online Rankings

Results from adjusted regression analyses (see Table 3) revealed that respondents with a chronic disease were nearly twice as likely to consult online rankings as respondents who were free of chronic disease (OR 2.09, 95% CI 1.66-2.63, *P*<.001). For levels of education, high school or less (OR 0.49, 95% CI 0.37-0.66, *P*<.001) and some college (OR 0.70, 95% CI 0.54-0.91, *P*=.01) were each associated with lower odds of consulting online rankings than respondents who had at least obtained a college degree. With respect to income, respondents who reported an annual income of US $75,000 to $150,000 were 1.5 times as likely to consult online rankings (OR 1.49, 95% CI 0.10-2.24, *P*=.05) compared to those making less than US $20,000. Males were less likely than females (OR 0.71, 95% CI 0.57-0.87, *P*<.001), whereas respondents who have a regular provider were more than 2 times more likely to consult online rankings (OR 2.05, 95% CI 1.52-2.78, *P*<.001). Living in an urban/suburban location was associated with a 60% increased chance of consulting rankings (OR 1.61, 95% CI 1.17-2.22, *P*<.001). In the adjusted model, marital status, race, and insurance coverage were not significantly associated with consulting online rankings. Likewise, the influence of having a health care provider and income was attenuated.

### Adjusted Regression Analyses for Using Social Networking Sites for Health

Adjusted odds ratios for using SNS for health-related purposes are presented in Table 4. As respondents’ ages increased, their likelihood for using such sites decreased (OR 0.96, 95% CI 0.95-0.97, *P*<.001). With respect to gender, males had lower odds than females (OR 0.70, 95% CI 0.56-0.87, *P*<.001). Compared to respondents without a regular health care provider, respondents with a regular provider had nearly twice the likelihood of using SNS for health-related activities (OR 1.89, 95% CI 1.43-2.52, *P*<.001), a greater influence than in the unadjusted model. Marital status was not significantly associated with using SNS for health-related activities.

**Table 1:** Sample characteristics (N=1745).

Characteristic		n (%)
**Gender**		
	Male	765 (43.84)
	Female	980 (56.16)
**Education**		
	Less than high school or high school graduate	546 (31.29)
	Some college	501 (28.71)
	College graduate or more	688 (39.43)
	Answer not give	10 (0.57)
**Income (US$)**		
	<$20,000	228 (13.07)
	$20,000 to <$40,000	347 (19.88)
	$40,000 to <$75,000	429 (24.58)
	$75,000 to <$150,000	514 (29.46)
	Don’t know or refused	227 (13.01)
**Race**		
	White	1382 (79.20)
	Black/African American	195 (11.17)
	All other races	136 (7.79)
	Answer not given	32 (1.83)
**Marital status**		
	Married or living with a partner	1060 (60.74)
	All other marital status	676 (38.74)
	Answer not given	9 (0.52)
**Geographic community type**		
	Rural	228 (13.07)
	Urban/suburban	1451 (83.15)
	Answer not given	66 (3.78)
**Has a chronic health condition**		
	Yes	699 (40.06)
	No	1046 (59.94)
**Has a regular health care provider**		
	Yes	1398 (80.11)
	No	343 (19.66)
	Answer not given	4 (0.23)
**Health insurance**		
	Yes	1516 (86.88)
	No	229 (13.12)
**Hispanic ethnicity**		
	Yes	182 (10.43)
	No	1552 (88.94)
	Answer not given	11 (0.63)
**Post health-related comments or questions on 5 social media**		
	Yes	265 (15.19)
	No	1480 (84.81)
**Use social networking sites for health-related information**		
	Yes	551 (31.58)
	No	1194 (68.42)
**Consulted online rankings or reviews of doctors, hospitals, drugs, or medical treatment**		
	Yes	718 (41.15)
	No	1027 (58.85)
**Posted a review of doctors, hospitals, drugs, or medical treatment**		
	Yes	173 (9.91)
	No	1572 (90.09)

^a^ The 5 social media are online discussion, listserv or other online group forum, a blog, a social networking site, Twitter or another status update site, and a website of any kind.

**Table 2:** Correlates of consulting online rankings or reviews (n=718) and use of social networking sites for health (n=551).

Sociodemographic and health characteristics		Total sample^a ^(N=1745)	Consulted online rankings or reviews		Use social networking sites for health	
			n (%)	*P*	n (%)	*P*
**Ethnicity**				.20		.26
	Hispanic/Latino	183	67 (36.61)		51 (27.87)	
	Non-Hispanic/Latino	1552	644 (41.49)		499 (32.15)	
**Education**				<.001		.21
	Less than high school diploma or a high school graduate	546	172 (31.50)		160 (29.30)	
	Some college	501	204 (40.71)		156 (31.14)	
	College degree or more	689	339 (49.37)		234 (33.96)	
**Chronic health condition**				<.001		.13
	Yes	699	347 (49.64)		206 (29.47)	
	No	1046	371 (35.47)		345 (32.95)	
**Income (US$)**				<.001		.03
	<$20,000	228	76 (33.33)		65 (28.51)	
	$20,000 to <$40,000	347	131 (37.75)		119 (34.29)	
	$40,000 to <$75,000	429	167 (38.93)		149 (34.73)	
	$75,000 to <$150,000	514	255 (49.61)		165 (32.10)	
	Don’t know/refused	227	88 (38.77)		54 (23.79)	
**Gender**				.001		.001
	Male	765	280 (36.60)		215 (28.10)	
	Female	979	437 (44.64)		335 (34.22)	
Age				<.001		<.001
**Geographic community type**				.001		.09
	Rural	229	75 (32.75)		61 (26.64)	
	Urban/suburban	1451	616 (42.45)		468 (32.25)	
**Health insurance**				.04		.64
	Yes	1516	654 (43.14)		475 (31.33)	
	No	229	64 (27.95)		75 (32.75)	
**Race**				.04		.52
	White	1382	588 (42.55)		444 (32.13)	
	Black/African American	195	68 (34.87)		63 (32.31)	
	All other races	136	47 (34.56)		37 (27.21)	
**Marital status**				.001		< .001
	Married or living with a partner	1060	468 (44.15)		284 (26.79)	
	All other marital status	676	244 (36.09)		264 (39.05)	
**Personal or family doctor or health care professional**				<.001		.047
	Yes	1398	632 (45.21)		457 (32.69)	
	No	343	83 (24.20)		93 (27.11)	

^a^ Total n may not equal 1745 due to missing values and rounding of weighted data.

**Table 3:** Consulted online rankings or reviews of doctors, hospitals, drugs, or medical treatments.

Sociodemographic and health characteristics		Unadjusted regression			Adjusted regression		
		Odds ratio (SE)	95% CI	*P*	Odds ratio (SE)	95% CI	*P*
**Chronic disease**							
	Does not have a chronic disease	1.0			1.0		
	Has a chronic disease	1.79 (0.10)	1.48-2.18	<.001	2.09 (0.10)	1.66-2.63	<.001
Age		0.96 (0.00)		<.001	0.99 (0.00)	0.98-0.10	.01
**Marital status**							
	Married	1.0			1.0		
	All other marital status	0.72 (0.10)	0.59-0.87	<.001	0.87 (0.13)	0.68-1.10	.26
**Education**							
	Less than high school graduate or high school graduate	0.48 (0.12)	0.38-0.60	<.001	0.49 (0.14)	0.37-0.65	<.001
	Some college	0.71 (0.012)	0.56-0.89	<.001	0.70 (0.13)	0.54-0.91	.01
	College degree or higher	1.0			1.0		
**Income (US$)**							
	<$20,000	1.0			1.0		
	$20,000 to <$40,000	1.21 (0.18)	0.85-1.72	0.28	1.20 (0.20)	0.82-1.79	.15
	$40,000 to <$75,000	1.28 (0.17)	0.91-1.79	.15	1.07 (0.20)	0.73-1.58	.72
	$75,000 to <$150,000	1.98 (0.17)	1.43-2.73	<.001	1.49 (0.21)	0.10-2.24	.05
	Don’t know/refused	1.27 (0.20)	0.87-1.87	.21	1.09 (0.23)	0.70-1.71	.70
**Gender**							
	Female	1.0			1.0		
	Male	0.72 (0.10)	0.59-0.87	<.001	0.71 (0.11)	0.57-0.87	<.001
**Geographic location**							
	Urban/suburban	1.59 (0.15)	1.13-2.04	.01	1.61 (0.16)	1.17-2.22	<.001
	Rural	1.0			1.0		
**Race**							
	White	1.0			1.0		
	Black/African American	0.72	0.52-0.98	.04	0.79 (0.18)	0.55-1.12	.19
	All other races	0.70	0.49-1.02	.06	0.69 (0.21)	0.46-1.03	.075
**Insurance coverage**							
	Does not have health insurance	1.0			1.0		
	Has health insurance	1.95 (0.16)	1.44-2.65	<.001	1.16 (0.18)	0.81-1.65	.41
**Regular health care provider**							
	Does not have a regular health care provider	1.0			1.0		
	Has a regular health care provider	2.59 (0.14)	1.98-3.39	<.001	2.05 (0.15)	1.52-2.78	<.001

**Table 4:** Used social networking sites for health-related activities, such as getting information, joining a group, following friends’ health experiences, raising money, increasing awareness, and remembering or memorializing others.

Sociodemographic and health characteristics		Unadjusted			Adjusted		
		Odds ratio (SE)	95% CI	*P*	Odds ratio (SE)	95% CI	*P*
Age		0.96 (0.00)	0.96-0.97	<.001	0.96 (0.00)	0.95-0.97	<.001
**Marital status**							
	Married	1.0			1.0		
	All other marital statuses	1.45 (0.11)	1.42-2.15	<.001	1.18 (0.12)	0.94-1.49	.16
**Gender**							
	Female	1.0			1.0		
	Male	0.75 (0.11)	0.61-0.93	.01	0.70 (0.11)	0.56-0.87	<.001
**Regular health care provider**							
	Does not have a regular health care provider	1.0			1.0		
	Has a regular health care provider	1.31 (0.13)	1.00-1.70	.048	1.89 (0.15)	1.43-2.52	<.001

## Discussion

This study examined the frequency of engaging in content creation through posting on social media sites, consumption of online rankings and reviews, and use of SNS for health-related activities. In addition, correlates for engaging in those behaviors were examined. The rate of online health information seeking behavior was similar to what has been reported previously [1,2]. Results show that although social media technologies allow people the opportunity to participate in the creation of online information, this is not very common; less than 15% of people reported doing so. In contrast, people are more likely to consume content with 30% to 40% of respondents reporting use of SNS for health-related activities and use of online rankings or review of doctors, hospitals, and medical treatments.

The lack of creating and contributing content is an intriguing finding. The value of social media is in the sharing of information within social networks. The rate of contributing opinions and experiences on other social media venues, such as product reviews or rankings sites (eg, Amazon or TripAdvisor), is similar to what we found in the current study [22]. People are not contributors. One explanation might be related to the fact that the frequency of encounters with doctors, hospitals, or medical treatments is less often, so there may be less motivation to share experiences. It could also be due to users’ feelings of incompetence relating to health topics, preferring to leave such discussions to trained professionals. Overall, there is a need for more research to understand the motivations and perceived benefits of contributing to health-related online forums, discussion boards, rating sites, and other social media venues.

Use of SNS for health was more common among females and younger people. These findings are not surprising given that this same group is more likely to use SNS in general [11]. Although growing in popularity among older populations, SNS use is still more common among people younger than 50 years of age, and particularly among the 18-29 year age group [11], which is consistent with our findings that younger audiences are more likely to use SNS for health-related activities.

People with chronic disease were twice as likely to consult online rankings or reviews. Previous research has shown that people in poorer health are more likely to seek health information online [6,23], hence consulting online data about doctors, hospitals, and medical treatments is probably reflective of the need for information to manage their condition. In addition, people who more frequently use health care services may be more invested in their health and, therefore, seek high quality experiences. Additionally, individuals with a chronic disease may also have greater medical knowledge about their condition and may feel more competent sharing that knowledge in a social media venue. It may be that use of social media for health is most applicable for specific segments of the population, such as those people who are trying to manage a chronic health condition.

Higher income was also associated with increased likelihood of consulting online rankings and reviews. This is similar to research that found health information seeking was also more common among higher income groups [24]. This may be because people with higher income use the Internet more often [25]. Alternatively, more health care options may be available to those with higher income than to those with lower income because of health insurance coverage, so they are able to discriminate among their choices in providers and treatment.

Regression models showed that having a regular health care provider is the only significant variable associated with both consulting online rankings and reviews and for using SNS for health-related activities. This is consistent with previous research that found that having a health care provider is associated with tracking personal health information on the Internet [26]. It might also be an artifact of higher income; individuals with a regular health care provider may also be wealthier. But the findings are inconsistent with Chou et al [17] who found that having a health care provider was not associated with social media use for health-related purposes, primarily due to age.

Variables that are traditionally associated with online health-seeking behavior, including race, geography, health insurance coverage, marital status, and education were not significant in terms of consulting online rankings for reviews or using SNS for health, both a type of health-seeking behavior. Thus, using social media for health seeking may be less influenced by common sociodemographic variables and may be better explained by other factors. For example, research on social network use has shown that personality traits, such as extroversion and neuroticism, are associated with social media use and sharing of information [27,28].

### Limitations

The data should be interpreted with caution considering the following data limitations. The internal consistency for the scales use to measure social media use ranged from .609 to .798. Although these are acceptable values according to conventional research standards, the individual items may not accurately capture the array of health behaviors one may engage in while using social media. For instance, people may be posting reviews online about other health-related experiences than the 3 assessed by this survey. Adding more variables would increase the internal consistency. These variables would have to be added to the Pew survey and may include items such as posting or consulting reviews of community or nonprofit facilities where services were received, using social networking sites to track personal progress toward health-related goals, or to receive social support, and so forth.

### Conclusions

People are using social media for seeking health information. However, individuals are more likely to consume information than they are to contribute to the dialog. The inherent value of “social” in social media is not being captured with online health information seeking. People with a regular health care provider, chronic disease, and those in younger age groups are more likely to consult online rankings and reviews and use SNS for health-related activities.

## Abbreviations

SNS: social networking sites
